# Blunt Cardiac Injury in the Severely Injured – A Retrospective Multicentre Study

**DOI:** 10.1371/journal.pone.0131362

**Published:** 2015-07-02

**Authors:** Marc Hanschen, Karl-Georg Kanz, Chlodwig Kirchhoff, Philipe N. Khalil, Matthias Wierer, Martijn van Griensven, Karl-Ludwig Laugwitz, Peter Biberthaler, Rolf Lefering, Stefan Huber-Wagner, TraumaRegister DGU

**Affiliations:** 1 Department of Trauma Surgery, Klinikum rechts der Isar, Technical University Munich, Munich, Germany; 2 Department of General, Visceral-, Transplantation-, Vascular- and Thoracic Surgery—Campus Grosshadern, University Hospital Munich (LMU), Munich, Germany; 3 I. Medical Department, Cardiology, Klinikum rechts der Isar, Technical University Munich, Munich, Germany; 4 IFOM–Institute for Research in Operative Medicine, University Witten/Herdecke, Faculty of Health, Cologne, Germany; 5 Committee on Emergency Medicine, Intensive Care and Trauma Management of the German Trauma Society (Sektion NIS), Berlin, Germany; Scuola Superiore Sant'Anna, ITALY

## Abstract

**Background:**

Blunt cardiac injury is a rare trauma entity. Here, we sought to evaluate the relevance and prognostic significance of blunt cardiac injury in severely injured patients.

**Methods:**

In a retrospective multicentre study, using data collected from 47,580 patients enrolled to TraumaRegister DGU (1993-2009), characteristics of trauma, prehospital / hospital trauma management, and outcome analysis were correlated to the severity of blunt cardiac injury. The severity of cardiac injury was assessed according to the abbreviated injury score (AIS score 1-6), the revised injury severity score (RISC) allowed comparison of expected outcome with injury severity-dependent outcome. N = 1.090 had blunt cardiac trauma (AIS 1-6) (2.3% of patients).

**Results:**

Predictors of blunt cardiac injury could be identified. Sternal fractures indicate a high risk of the presence of blunt cardiac injury (AIS 0 [control]: 3.0%; AIS 1: 19.3%; AIS 2-6: 19.1%). The overall mortality rate was 13.9%, minor cardiac injury (AIS 1) and severe cardiac injury (AIS 2-6) are associated with higher rates. Severe blunt cardiac injury (AIS 4 and AIS 5-6) is associated with a higher mortality (OR 2.79 and 4.89, respectively) as compared to the predicted average mortality (OR 2.49) of the study collective.

**Conclusion:**

Multiple injured patients with blunt cardiac trauma are at high risk to be underestimated. Careful evaluation of trauma patients is able to predict the presence of blunt cardiac injury. The severity of blunt cardiac injury needs to be stratified according to the AIS score, as the patients’ outcome is dependent on the severity of cardiac injury.

## Introduction

According to the Centers for Disease Control and Prevention (www.cdc.gov), trauma (accidents / unintentional injuries) remains to be the leading cause of death in the United States up to the age of 44 years. About 25% of patients of all traumatic fatalities represent blunt thoracic trauma [1]. Although blunt cardiac trauma is a known complication, not much is known about the incidence, the relevance and the outcome of this trauma entity. The reported incidence of cardiac damage following blunt chest trauma ranges between 8% and 76%, depending on the criteria used to diagnose [2–6]. The literature is inconsistently interpreting the little data available in terms of relevance and prognostic significance. While some studies indicate that blunt cardiac trauma is rare and has little prognostic significance [7,8], others show that it is affecting the patients’ outcome [9].

The final common path in the pathology of blunt cardiac trauma is injury to the myocardium, additional injury to anatomical structures such as the valves are facultative. There are two basic mechanisms leading to blunt cardiac trauma: direct impact against bony structures (sternum, vertebrae) and shear stress forces [10,11]. They result in cellular damage with necrosis, permeation of blood cells into the myocardial tissue and subsequent scar formation [10]. Due to cellular damage and disruption of myocardial cell membranes, creatine phosphokinase myocardial band (CPK-MB) and troponin (troponin I and T) are released [12]. Injuries to anatomical structures might involve laceration or rupture of valves, due to the higher pressure the left side is more susceptible to injury of anatomical structures (mitral and aortic valve) as compared to the right side (tricuspid and pulmonic valve) [11]. In addition, injury to coronary arteries or to the septum may occur.

The diagnosis of blunt cardiac trauma is challenging, visible thoracic lesions might be absent. In addition, clinical signs such as hypotension, hypoxia or haemodynamic instability might be attributed to other severe injuries with blood loss in trauma patients [13]. Patients might present with palpitations or precordial pain, which are often misinterpreted as concomitant muscle injury [14]. The use of biochemical biomarkers is neither plain sailing. CPK-MB has a high specificity for myocardial infarction, but due to high increases of CPK following trauma, false positive increases of CPK-MB were found in severely injured patients [4,10]. In contrast, troponin I and T are highly specific for myocardial injury, normal concentrations strongly indicate the absence of myocardial injury following blunt chest trauma [15–17]. Electrocardiography (ECG) might show non-specific abnormalities or might be normal following trauma due to anatomical reasons [13]. The relative frequency of injury to the heart is as follows: right ventricle, left ventricle, right atrium, intraventricular septum, left atrium, and least commonly rupture of the intra-atrial septum. Right heart injury might be missed on an ECG, as the ECG is more sensitive for left ventricular than for right ventricular injuries due to the muscle mass ratio [18]. In contrast to ECG, echocardiography enables the clinician to localise myocardial wall dysfunction. In addition to the determination of wall motion abnormalities, anatomical injuries to the heart can be determined (e.g. valvar lesions or pericardial tamponade) [19,20]. Echocardiography is thereby a valuable tool to diagnose blunt cardiac trauma. Radionuclide imaging such as SPECT or PET are not recommended due to poor visualisation especially of the right ventricle and insufficient experience of the technique in the trauma setting [2].

Defining the severity of blunt cardiac trauma remains challenging, the spectrum ranges from minor ‘bruises’ to penetrating wounds including more than 50% of a chamber. The Association for the Advancement of Automotive Medicine (AAAM) created an anatomical-based coding system describing the severity of injuries, called Abbreviated Injury Score (AIS). First published in 1969, multiple updates have been implemented. Being the gold standard tool to scale single injuries, the AIS scales injury from 1–6 (1 minor, 2 moderate, 3 serious, 4 severe, 5 critical, and 6 maximum).

While diagnostic strategies have been refined in the past, little data concerning the relevance of blunt cardiac injury in the context of outcome, prognosis and mortality has been published. In addition, not much is known about predictors of blunt cardiac injury. To approach these essential questions, we collected data from the TraumaRegister DGU (1993–2009), which were retrieved according to the AIS score. Data of 47,580 patients were included to investigate the relevance and prognostic significance of blunt cardiac trauma in severely injured patients. Here, we emphasize the relevance of blunt cardiac trauma and its relevance concerning the outcome of severely injured patients, thereby sensitising trauma surgeons to this rare trauma entity.

## Methods

### Data collection

Data was obtained from the TraumaRegister DGU of the German Trauma Society (DGU). The TraumaRegister DGU was initiated in 1993 by the Committee on Emergency Medicine, Intensive Care and Trauma Management of the German Trauma Society (Sektion NIS) and comprises data of severely injured patients of more than 600 trauma centres. Participating hospitals are mainly located in Germany (90%), a rising number of hospitals of other countries contributes as well (Austria, Belgium, China, Finland, Luxembourg, Slovenia, Switzerland, The Netherlands, and the United Arab Emirates). It is a prospective, multicentric, standardised and anonymised data base. The inclusion criterion to the TraumaRegister DGU is admission to the hospital via emergency room with subsequent ICU/ICM care or admission to the hospital with vital signs and death before admission to ICU. Data are continuously entered into a web-based data server that is hosted by the German Trauma Society and its AUC—Academy for Trauma Surgery. Data anonymity is double-sided, thereby guaranteed for the individual patients and the participating hospitals. The registry collects epidemiologic, physiologic, laboratory, diagnostic, operative, interventional and intensive care medical data as well as scoring and outcome data.

We used data entered between 1993 and 2009. Cardiac trauma was identified and graded according to the abbreviated injury score, version 2005 (AIS codes 4404xx.x, 4410xx.x, 4412xx.x, 4413xx.x, 4416xx.x, 4208xx.x). Unfortunately, minor blunt cardiac trauma is not well-reflected by the AIS, even apparently small injury to the heart is being scored with an AIS of 3. For example, minor injury to the heart as reflected by abnormal CPK-MB/ troponin T and minor ECG abnormality without any signs of cardiac failure is graded with an AIS of 3, while blunt cardiac injury with signs of cardiac failure is attributed an AIS of 3 as well. Mattox and co-workers therefore suggested to score the AIS down in certain cases [21]. To allow for sub-analysis we therefore modified the score in one detail. The AIS codes 441099.3, 441002.3, and 441004.3 (all minor contusion of the myocardium) were scored down to AIS 1. This is in line with the above-mentioned recommendation and with the scale of Moore and co-workers [22]. The correlation to the AIS-2005 score and the scale of Moore et al. as well as our modification to the AIS score is being shown in Table 1.

**Table 1:** Heart Injury Scale.

Grade*	Description of Injury	AIS code	AIS code modification
I	Blunt cardiac injury with minor ECG abnormality (nonspecific ST or T wave changes, premature arterial or ventricular contraction or persistent sinus tachycardia)	441099.3	441099.1
	Blunt or penetrating pericardial wound with out cardiac injury, cardiac tamponade, or cardiac herniation	441002.3	441002.1
		441004.3	441004.1
II	Blunt cardiac injury with heart block (right or left bundle branch, left anterior fascicular, or atrioventricular) or ischemic changes (ST depression or T wave inversion) without cardiac failure	441699.2	
	Penetrating tangential myocardial wound up to, but not extending through endocardium, without tamponade	441602.2	
III	Blunt cardiac injury with sustained (>6 beats/min) or multilocal ventricular contractions	441008.3	
	Blunt or penetrating cardiac injury with septal rupture, pulmonary or tricuspid valvular incompetence, papillary muscle dysfunction, or distal coronary arterial occlusion without cardiac failure	441010.3	
	Blunt pericardial laceration with cardiac herniation	441604.3	
	Blunt cardiac injury with cardiac failure		
	Penetrating tangential myocardial wound up to, but extending through, endocardium, with tamponade		
IV	Blunt or penetrating cardiac injury with septal rupture, pulmonary or tricuspid valvular incompetence, papillary muscle dysfunction, or distal coronary arterial occlusion producing cardiac failure	441006.4	
	Blunt or penetrating cardiac injury with aortic mitral valve incompetence		
	Blunt or penetrating cardiac injury of the right ventricle, right atrium, or left atrium		
V	Blunt or penetrating cardiac injury with proximal coronary arterial occlusion	440400.5	
	Blunt or penetrating left ventricular perforation	441012.5	
	Stellate wound with < 50% tissue loss of the right ventricle, right atrium, or of left atrium	441606.5	
VI	Blunt avulsion of the heart; penetrating wound producing > 50% tissue loss of a chamber	441018.6	

Scaling system for heart injuries, as described by Moore et al. [22]. Correlation to the Abbreviated Injury Score (AIS) 2005 is given. To account for the recommendation of Mattox et al. [21], minor blunt injury was scaled down (see AIS modification).

*Advance one group for multiple wounds to a single chamber or multiple chamber involvement.

A total number of 51,425 cases were identified within the collection time, the dataset was adjusted for incomplete data and for penetrating injuries, thereby resulting in 47,580 cases. Blunt cardiac injury was diagnosed in 1,090 cases (AIS-heart 1–6, 2.3%), 46,490 cases did not include cardiac injury (AIS 0) and served as severely injured control patients.

The RISC score was applied to calculate the expected mortality following blunt cardiac trauma (odds ratio [OR]). Details on the RISC score can be found in previous studies [23]. Briefly, the RISC score was developed by the Institute for Research in Operative Medicine aiming to calculate the risk of death in injured patients. Developed with a dataset of the TraumaRegister DGU from 1993–2000 including 2,009 patients, the RISC score has been shown to be equipped with a significantly better goodness of fit according to Hosmer and colleagues [24] as compared to other mortality-predicting scores, e.g. the trauma and injury severity score (TRISS) [25].

The present study is in line with the publication guidelines of the TraumaRegister DGU and registered as TR-DGU project ID 2010–013. Patient information was anonymized and de-identified prior to analysis, irreversible data anonymity is guaranteed both for the individual patients and the participating hospitals. As the data in the TraumaRegister DGU are anonymised and routinely collected clinical data obtained from the patients chart no written consent was given by the patients. This has been waived by the approving ethics committee of the medical faculty of Technical University Munich (TUM), Germany (Project number 15/15).

### Statistical Analysis

The descriptive data analysis was performed using the χ^2^ test and t-test (both two-sided). The RISC score- and AIS-specific odds ratio was calculated for outcome analysis. Details on the methodology were described previously [23]. Statistical analysis was done using SPSS version 18.0. If not declared otherwise, p<0.05 is considered statistically significant.

## Results

### Characteristics

A total number of 47,580 patients were included in this study (Table 2). N = 1.090 had blunt cardiac trauma (AIS 1–6), representing 2.3% of patients included. The mean age of the collective was 42.7 years, about 72% of the patients were male. The mean injury severity score (ISS) for the above-mentioned collective was 22.51 ± 13.9 (mean ± SD). The most common cause for blunt cardiac injury were road traffic accidents, attributing for 60.5% of all blunt cardiac trauma cases. In the prehospital setting, 28.4% of the total number of patients were found unconscious (GCS<8) and 17.5% in shock. Interestingly, patients with a severity of blunt cardiac injury ranging from AIS 2–6 were found unconscious in 46.3% of all cases, and in 39.3% of all cases in shock. In addition to unconsciousness and shock, resuscitation on scene was strongly correlated to the severity of injury. Blunt cardiac injury with an AIS 2–6 was associated with the need for cardiac resuscitation in about 20% of all cases, while the overall resuscitation rate was about 3%. Chest drains were placed on scene in about 5.4% of all cases. Again, severity of injury is associated with an increased need for intervention. Cases with an AIS of 1 had chest drains placed in 12.5% of all cases, cases with an AIS of 2–6 had chest drains placed in 18.8% of all cases on scene.

**Table 2:** Characteristics.

	AIS 0	AIS 1	AIS 2–6	Total	p value
**Age**	42.72 ± 21.17	41.13 ± 19.69	46.20 ± 21.50	42.71 ± 21.15	0.004
**Men**	33207 / **46269**	661 / **861**	160 / **222**	34028 / **47352**	0.005
	71.80%	76.80%	72.10%	71.90%	
**Prehospital**					
**Road traffic accident**	27713 / **46055**	660 / **860**	166 / **223**	28539 / **47138**	<0.001
	60.20%	76.70%	74.40%	60.50%	
**GCS < 8**	10830 / **38285**	222 / **726**	87 / **188**	11139 / **39199**	<0.001
	28.30%	30.60%	46.30%	28.40%	
**Shock on scene (SBP < 90 mmHg)**	6185 / **35723**	161 / **668**	66 / **168**	6412 / **36559**	<0.001
	17.30%	24.10%	39.30%	17.50%	
**Chest drain on scene**	1959 / **37892**	91 / **726**	36 / **191**	2086 / **38809**	<0.001
	5.20%	12.50%	18.80%	5.40%	
**Resuscitation (CPR) on scene**	1144 / **37892**	30 / **726**	38 / **191**	1212 / **38809**	<0.001
	3.00%	4.10%	19.90%	3.10%	
**Trauma room / Hospital**					
**Shock on admission (SBP < 90 mmHg)**	4976 / **42169**	152 / **804**	75 / **200**	5203 / **43173**	<0.001
	11.80%	18.90%	37.50%	12.10%	
**ICU admission**	40770 / **46490**	806 / **865**	173 / **225**	41749 / **47580**	<0.001
	87.70%	93.20%	76.90%	87.70%	
**MOF**	8157 / **37811**	291 / **736**	83 / **142**	8531 / **38689**	<0.001
	21.60%	39.50%	58.50%	22.10%	
**Sepsis**	3175 / **37918**	123 / **720**	37 / **144**	3335 / **38782**	<0.001
	8.40%	17.10%	25.70%	8.60%	
**Massive blood transfusion (>10 PRBC)**	2589 / **46158**	76 / **861**	43 / **217**	2708 / **47236**	<0.001
	5.60%	8.80%	19.80%	5.70%	
**Chest drain on admission**	7150 / **42243**	279 / **796**	97 / **193**	7526 / **43232**	<0.001
	16.90%	35.10%	50.30%	17.40%	
**Resuscitation (CPR) on admission**	1501 / **42243**	52 / **796**	44 / **193**	1597 / **43232**	<0.001
	3.60%	6.50%	22.80%	3.70%	
**Thoracotomy**	0 / **46490**	47 / **865**	60 / **225**	107 / **47580**	<0.001
	0.00%	5.40%	26.70%	0.20%	
**Sternal fracture**	1406 / **46490**	167 / **865**	43 / **225**	1616 / **47580**	<0.001
	3.00%	19.30%	19.10%	3.40%	
**Mortality rate**					
**24h**	3307 / **46490**	68 / **865**	70 / **225**	3445 / **47580**	<0.001
	7.10%	7.90%	31.10%	7.20%	
**Overall**	6401 / **46490**	138 / **865**	97 / **225**	6636 / **47580**	<0.001
	13.80%	16.00%	43.10%	13.90%	

The characteristics of the 47,580 patients included to this study are given. AIS Abbreviated Injury Score, GCS Glasgow Coma Scale, SBP Systolic Blood Pressure, ICU Intensive Care Unit, MOF Multiple Organ Failure, PBRC Packed Red Blood Cells, p: χ^2^ test or Mann-Whitney-U test (two-sided).

Comparable to the prehospital setting, certain characteristics were evident in analysing data from the trauma room management and the hospital care. Again, more severe blunt cardiac injury, as defined by AIS 2–6, were associated with a significant increase in the rate of massive blood transfusion (19.8%), resuscitation on admission (22.8%), and need for thoracotomy (26.7%). In contrast, less severe injury (AIS 1) cases were observed to need less massive blood transfusion (8.8%), less resuscitation on admission (6.5%), and lower need for thoracotomy (5.4%). Interestingly, sternal fracture is evident in one fifth of all cases even in mild severity of blunt cardiac injury (AIS 1). The impact of undergoing blunt cardiac injury is being underlined by high frequencies of ICU admissions and multiple organ failure / sepsis even in cases of AIS 1.

The 24h mortality is highest for cases with an injury severity of AIS 2–6, reaching about 31.1%. The total frequency of 24h mortality is at about 7.2%. Interestingly, as compared to the 24h mortality (7.9%), the overall mortality for AIS 1 cases reaches 16.0%, thereby being double as high. The overall mortality for AIS 2–6 cases (43.1%) is not increasing to this extent as compared to the 24h figure (31.1%).

### Injury Scores

As by definition, an injury severity score ≥ 16 resembles the prevalence of a multiple injured patient. In more than 80% of cases with an AIS of 1 and higher, the definition of multiple injured patients are met (Table 3). The most common injury pattern is mainly restricted to combinations of blunt heart injury with head trauma or with thorax trauma, reaching frequencies of more than 40%.

**Table 3:** Injury Severity Score and Abbreviated Injury Score (AIS).

	AIS 0	AIS 1	AIS 2–6	Total	p value
**Injury severity score ≥ 16**	31361 / **46490**	692 / **865**	214 / **225**	32267 / **47580**	<0.001
	67.50%	80.00%	95.10%	67.80%	
**Abbreviated injury scale (≥ 3)**					
**Head**	21346 / **46490**	350 / **865**	98 / **225**	21794 / **47580**	<0.005
	45.90%	40.50%	43.60%	45.80%	
**Thorax**	19084 / **46490**	665 / **865**	217 / **225**	19966 / **47580**	<0.001
	41.00%	76.90%	96.40%	42.00%	
**Abdomen**	7245 / **46490**	195 / **865**	72 / **225**	7512 / **47580**	<0.001
	15.60%	22.50%	32.00%	15.80%	
**Extremities**	14818 / **46490**	317 / **865**	87 / **225**	15222 / **47580**	<0.001
	31.90%	36.60%	38.70%	32.00%	

ISS ≥ 16 defines the presence of a severely injured patient. p: χ^2^ test or Mann-Whitney-U test (two-sided).

### Glasgow Outcome Scale

Up to 47.9% of all cases had a low disability outcome, 24.5% a moderate disability outcome (Table 4). The overall mortality rate is 14.5% (note: the GOS mortality rate is not equal to the overall mortality rate of Table 2 due to the fact that GOS has not been documented for all patients). Notably, while low injury severity (AIS 1) was associated with mortality rates of 15.6%, the overall mortality rate is at 45.1% for cases with an injury severity of AIS 2–6.

**Table 4:** Glasgow Outcome Scale.

	AIS 0	AIS 1	AIS 2–6	Total	p value
**Low Disability**	16237 / 33781	313 / 675	45 / 184	16595 / 34640	<0.001
	48.10%	46.40%	24.50%	47.90%	
**Moderate Diability**	8274 / 33781	166 / 675	32 / 184	8472 / 34640	<0.001
	24.50%	24.60%	17.40%	24.50%	
**Severe Disability**	3497 / 33781	75 / 675	18 / 184	3590 / 34640	<0.001
	10.40%	11.10%	9.80%	10.40%	
**Persistent vegetative state**	931 / 33781	16 / 675	6 / 184	953 / 34640	<0.001
	2.80%	2.40%	3.30%	2.80%	
**Death**	4842 / 33781	105 / 675	83 / 184	5030 / 34640	<0.001
	14.30%	15.60%	45.10%	14.50%	

p: χ^2^ test or Mann-Whitney-U test (two-sided).

### Multivariate Analysis

We conducted multivariate analysis to investigate the effect of blunt cardiac injury on trauma-specific mortality, scored according to the AIS (Table 5). Scores of AIS 4 and AIS 5/6 for blunt cardiac injury were the strongest predictors of trauma-associated mortality (AIS 4 OR 2.79 with 95% CI 1.45–5.41 and AIS 5/6 OR 4.89 with 95% CI 1.83–13.0). Notably, the recorded mortality for blunt cardiac injury rated AIS 1 and AIS 2/3 did not reach significance. The OR (AIS 1 OR 1.09 with 95% CI 0.85–1.40 and AIS 2/3 OR 0.81 with 95% CI 0.43/1.54) is well below the expected OR for patients following blunt cardiac injury, as calculated by the RISC score (OR 2.49 with 95% CI 2.44–2.54).

**Table 5:** Multivariate Analysis, injury-specific mortality.

	Odds Ratio	95% CI	P-value	Regression Coefficient
**RISC**	2.49	2.44–2.54	<0.001	0.91
**Heart injury**				
**AIS 0**	-	-	<0.001	-
**AIS 1**	1.09	0.85–1.40	0.484	0.08
**AIS 2 / 3**	0.81	0.43–1.54	0.812	0.21
**AIS 4**	**2.79**	**1.45–5.41**	**0.002**	**1.03**
**AIS 5 / 6**	**4.89**	**1.83–13.0**	**0.002**	**1.59**

RISC Revised Injury Severity Classification, CI Confidence Interval. Significant predictors are in bold type.

## Discussion

In our analysis, blunt cardiac injury was diagnosed in 1,090 cases (2.3%). To the best of our knowledge, this is one of the most extensive studies in this field. The reported incidence of cardiac injury following closed blunt chest trauma ranges between 8% an 76%, depending on the definition and tests for cardiac injury [2]. In autopsy studies following major blunt trauma, an incidence of cardiac contusion ranging from 14%-16% has been reported [6]. As shown in our study, blunt cardiac injury is associated with an early mortality of 7.2% and with an overall mortality of 13.9%. These figures are in line with previous findings reported in the literature, ranging from 0–17% overall survival rate [26,27]. According to the literature, cardiac injury is the most overlooked injury in patients who die from trauma [28]. The high incidence of blunt cardiac injury in conjunction with the unfavourable outcome as reflected by the high mortality rate finds itself in a surprising contrast to the knowledge of this injury pattern. The quote by Burchell in 1935 ‘And always with a heart contusion arise both doubt and much confusion’ [29] is still valid in our days. This manuscript is the first attempt in a large-scale approach to characterise this entity of heart disease and to present predictors of its presence and outcome.

Blunt cardiac trauma, irrespective of its severity, can most frequently be encountered in male patients between 30 and 50 years of age. It is most commonly caused by the combination of deceleration forces, compression forces, and shearing stress [30]. This triad can frequently be encountered due to road traffic accidents, as could be identified for up to 60.5% of the patients included in this study. Interestingly, cardiac injury due to deceleration forces has been reported after deceleration from velocities of less than 20 mph [10]. Compression forces can result from abdominal and lower extremity trauma as well, often referred to as ‘hydraulic ram effect’ [18,31]. In the prehospital setting, unconsciousness (GCS <8), shock, need for chest drain placement, and CPR on scene strongly indicate blunt cardiac injury. The presence of these characteristics should raise suspicion for blunt cardiac injury in the prehospital setting, furthermore they correlate with the severity of blunt cardiac injury.

Thorough clinical examination is a prerequisite in assessing the presence and severity of blunt cardiac injury. In approximately 75% of patients diagnosed with blunt cardiac injury, thoracic concomitant injuries can be diagnosed [32]. These include for example pneumothorax, haemothorax, flail chest, rib fractures, and sternal fractures. According to the data analysed in this study, the presence of sternal fractures is strongly indicative for blunt cardiac injury. Interestingly, not only severe cardiac blunt injury (AIS 2–6) is associated with a high incidence of sternal fractures, but in minor blunt cardiac injury (AIS 1) sternal fractures can also be diagnosed in up to 20% of the patients. Our results identify sternal fractures as a strong indicator of blunt cardiac injury. In our opinion, the presence of a sternal fracture should therefore be followed by specific diagnostics and algorithm-based management. In contrast to our findings, others studies suggest no relationship between sternal fractures and blunt cardiac injury [33]. Interestingly, the cited group proposes that no specific diagnostics or management are needed in patients with sternal fractures–which in our opinion is to be considered risky against the background of our results.

Depending on the severity of blunt cardiac injury, concomitant injuries are more frequent and more severe. On average, the AIS scores for concomitant injuries of the head, the thorax, the abdomen, or the extremities are highest in the presence of cardiac injury scaled AIS 2–6.

According to our data, about 87% of patients have been admitted to a monitored bed for observation (intensive care unit). No differences could be detected in this course of action between patients with an injury severity of AIS 1 –AIS 6. This is in line with findings in the literature. Even in the presence of minor abnormalities of the admission ECG, monitored bed observation is recommended [34]. This recommendation is reasoned by the fact that there is unfortunately no correlation between the complexity of arrhythmias and the degree of cardiac injury [2]. In blunt cardiac injury, about 40–80% of patients have abnormal ECGs [10,32]. The indication of ICU observation is met on a grand scale not least because diagnostic tools are limited and false negative results might mislead. Most trauma centres apply protocols including ECG, basic blood tests and cardiac enzymes, followed by serial ECGs, cTnI or cTnT to monitor changes or progression of the injuries [11]. We strongly recommend to obtain expert knowledge in every case of blunt cardiac injury: Management of these patients should be done in a team approach together with the cardiology department.

The effect of blunt cardiac injury on trauma-specific mortality was analysed using multivariate analysis. Using the RISC score, the probability of death following trauma was first calculated for all patients included in the study (resembling the average), the OR of 2.49 allows for interpretation of the data by comparison. Interestingly, the OR in patients with blunt cardiac trauma AIS 1–3 (*minor–moderate–serious*) ranging from 0.81–1.09 is significantly lower as compared to the average probability of death in the given population. Blunt cardiac trauma scaled AIS 4–6 (*severe–critical–maximum*) are on the other hand associated with a higher risk of death as compared to the average (OR 2.79–4.89). These findings underline the need to carefully distinguish blunt cardiac injury of different severities.

### Limitations

There are several limitations to our study. The analysis is based on retrospective collection of data. Due to missing data in the trauma register, aberrations in the number of total patients occurred for the Glasgow Outcome Scale and certain characteristics.

As mentioned above, the diagnosis of blunt cardiac injury is challenging. This analysis is based on a registry search, cardiac injury was identified in the participating hospitals and graded according to the abbreviated injury score (definition given in Table 1). The diagnosis of blunt cardiac injury was locally verified in the participating hospitals using standard measures like clinical parameters, serum markers, ECG, as well as echocardiography. Unfortunately, no cardiac enzyme serum markers (TnT; CPK-MB) or ECG information is included in the data set of the TraumaRegister DGU, retrospective validation of the identification of blunt cardiac injury could therefore not be performed. Results may be biased due to inhomogeneous standards in the treatment of severely injured patients. Whether all hospitals implemented the concept of advanced trauma life support remains unclear. In addition, grading of injuries might be biased due to potential inhomogeneous standards in the grading of injuries. Structural and geographical differences between regions and federal states were not considered. Finally, intercentre inconsistency (differences in equipment and staff training) might represent confounding factors which have to be taken into account.

### Conclusion and practical implications

About every 50^th^ multiple injured patient suffers, beside other injuries, from blunt cardiac trauma. We identified strong indicators for the presence of blunt cardiac injury. In the preclinical setting, unconsciousness (GCS <8), shock, need for chest drain placement, and CPR on scene should raise awareness of this injury pattern. In consideration of the reduction in postoperative complications and mortality following implementation of the WHO surgical safety checklist [35], we recommend screening checklists in emergency rooms, facilitating the identification of high risk patients for blunt cardiac injury according to the AIS score. Based on our findings, the screening checklist should incorporate preclinical and clinical parameters. Of note, treatment of patients in shock being non-responders should immediately arouse suspicion of blunt cardiac injury being the underlying cause. Our results raise awareness to carefully grade the severity of blunt cardiac injury. In any kind of doubt, 12-lead ECG should be performed. Further investigation should include echocardiography as part of the secondary survey according to ATLS (Advanced Trauma Life Support) [36]. The severity of blunt cardiac trauma, as could be shown, is correlated to the outcome and survival of trauma patients. Interestingly, concomitant injuries to other sites and organs are not correlated to the severity of blunt cardiac trauma. For example, sternal fractures indicate the presence of blunt cardiac injury irrespective of its severity. On the basis of our findings, we recommend screening for blunt cardiac injury in every case of thoracic trauma, especially in the presence of sternal fractures (indicator injury). The knowledge of the potential high impact of blunt cardiac trauma on patient survival should raise high alertness in the trauma team to this common kind of injury. Management of blunt cardiac injury should be performed in a team approach, cardiologists should be involved in any cases of doubt.

## References

[1] Wilson RF, Murray C, Antonenko DR. Nonpenetrating thoracic injuries. Surg Clin North Am 1977;57: 17–36. 854851 10.1016/s0039-6109(16)41131-x

[2] Feghali NT, Prisant LM. Blunt myocardial injury. Chest 1995;108: 1673–1677. 7497780 10.1378/chest.108.6.1673

[3] Dubrow TJ, Mihalka J, Eisenhauer DM, de Virgilio C, Finch M, Mena IG, et al Myocardial contusion in the stable patient: what level of care is appropriate? Surgery 1989;106: 267–273. 2763029

[4] Frazee RC, Mucha P Jr, Farnell MB, Miller FA Jr. Objective evaluation of blunt cardiac trauma. J Trauma 1986;26: 510–520. 3723617 10.1097/00005373-198606000-00004

[5] McCarthy MC, Pavlina PM, Evans DK, Broadie TA, Park HM, Schauwecker DS. The value of SPECT-thallium scanning in screening for myocardial contusion. Cardiovasc Intervent Radiol 1991;14: 238–240. 1913737 10.1007/BF02578469

[6] Wisner DH, Reed WH, Riddick RS. Suspected myocardial contusion. Triage and indications for monitoring. Ann Surg 1990;212: 82–86. 2363607 10.1097/00000658-199007000-00011 PMC1358077

[7] Beresky R, Klingler R, Peake J. Myocardial contusion: when does it have clinical significance? J Trauma 1988;28: 64–68. 3339665

[8] Fildes JJ, Betlej TM, Manglano R, Martin M, Rogers F, Barrett JA. Limiting cardiac evaluation in patients with suspected myocardial contusion. Am Surg 1995;61: 832–835. 7661485

[9] Baum V. Anesthetic complications during emergency noncardiac surgery in patients with documented cardiac contusions. J Cardiothorac Vasc Anesth 1991;5: 57–60. 1868186 10.1016/1053-0770(91)90095-b

[10] Tenzer ML. The spectrum of myocardial contusion: a review. J Trauma 1985;25: 620–627. 2989545 10.1097/00005373-198507000-00008

[11] Bansal MK, Maraj S, Chewaproug D, Amanullah A. Myocardial contusion injury: redefining the diagnostic algorithm. Emerg Med J 2005;22: 465–469. 15983078 10.1136/emj.2004.015339 PMC1726836

[12] Adams JE III, vila-Roman VG, Bessey PQ, Blake DP, Ladenson JH, Jaffe AS. Improved detection of cardiac contusion with cardiac troponin I. Am Heart J 1996;131: 308–312. 8579026 10.1016/s0002-8703(96)90359-2

[13] Sybrandy KC, Cramer MJ, Burgersdijk C. Diagnosing cardiac contusion: old wisdom and new insights. Heart 2003;89: 485–489. 12695446 10.1136/heart.89.5.485 PMC1767619

[14] Illig KA, Swierzewski MJ, Feliciano DV, Morton JH. A rational screening and treatment strategy based on the electrocardiogram alone for suspected cardiac contusion. Am J Surg 1991;162: 537–543. 1670221 10.1016/0002-9610(91)90105-m

[15] Salim A, Velmahos GC, Jindal A, Chan L, Vassiliu P, Belzberg H, et al Clinically significant blunt cardiac trauma: role of serum troponin levels combined with electrocardiographic findings. J Trauma 2001;50: 237–243. 11242287 10.1097/00005373-200102000-00008

[16] Collins JN, Cole FJ, Weireter LJ, Riblet JL, Britt LD. The usefulness of serum troponin levels in evaluating cardiac injury. Am Surg 2001;67: 821–825. 11565757

[17] Rajan GP, Zellweger R. Cardiac troponin I as a predictor of arrhythmia and ventricular dysfunction in trauma patients with myocardial contusion. J Trauma 2004;57: 801–808. 15514534 10.1097/01.ta.0000135157.93649.72

[18] El-Chami MF, Nicholson W, Helmy T. Blunt cardiac trauma. J Emerg Med 2008;35: 127–133. 17976783 10.1016/j.jemermed.2007.03.018

[19] Hiatt JR, Yeatman LA Jr, Child JS. The value of echocardiography in blunt chest trauma. J Trauma 1988;28: 914–922. 3398089 10.1097/00005373-198807000-00003

[20] Karalis DG, Victor MF, Davis GA, McAllister MP, Covalesky VA, Ross JJ Jr, et al The role of echocardiography in blunt chest trauma: a transthoracic and transesophageal echocardiographic study. J Trauma 1994;36: 53–58. 8295249 10.1097/00005373-199401000-00008

[21] Mattox KL, Flint LM, Carrico CJ, Grover F, Meredith J, Morris J, et al Blunt cardiac injury. J Trauma 1992;33: 649–650. 1464909

[22] Moore EE, Malangoni MA, Cogbill TH, Shackford SR, Champion HR, Jurkovich GJ, et al Organ injury scaling. IV: Thoracic vascular, lung, cardiac, and diaphragm. J Trauma 1994;36: 299–300. 8145307

[23] Huber-Wagner S, Lefering R, Qvick LM, Korner M, Kay MV, Pfeifer KJ, et al Effect of whole-body CT during trauma resuscitation on survival: a retrospective, multicentre study. Lancet 2009;373: 1455–1461. 10.1016/S0140-6736(09)60232-4 19321199

[24] Hosmer DW, Hosmer T, Le CS, Lemeshow S. A comparison of goodness-of-fit tests for the logistic regression model. Stat Med 1997;16: 965–980. 9160492 10.1002/(sici)1097-0258(19970515)16:9<965::aid-sim509>3.0.co;2-o

[25] Lefering R. Revised injury severity classification (RISC)-development and validation of a classification system for severely injured patients based on a large trauma registry. (Revised Injury Severity Classification (RISC)—Entwicklung und Validierung eines Schweregrad-Klassifikationssystems für schwerverletzte Patienten zur Anwendung in einem nationalen Traumaregister). PhD thesis,University Witten/Herdecke, 2007. Available: notesweb.dmz.uni-wh.de/public/UWHForschung.nsf/

[26] Rosemurgy AS, Norris PA, Olson SM, Hurst JM, Albrink MH. Prehospital traumatic cardiac arrest: the cost of futility. J Trauma 1993;35: 468–473. 8371308

[27] Battistella FD, Nugent W, Owings JT, Anderson JT. Field triage of the pulseless trauma patient. Arch Surg 1999;134: 742–745. 10401826 10.1001/archsurg.134.7.742

[28] Liedtke AJ, DeMuth WE Jr. Nonpenetrating cardiac injuries: a collective review. Am Heart J 1973;86: 687–697. 4582700 10.1016/0002-8703(73)90349-9

[29] Burchell HB. Unusual forms of heart disease. Circulation 1954;10: 574–579. 13209744 10.1161/01.cir.10.4.574

[30] Kaye P, O'Sullivan I. Myocardial contusion: emergency investigation and diagnosis. Emerg Med J 2002;19: 8–10. 11777862 10.1136/emj.19.1.8 PMC1725746

[31] Cooper GJ, Pearce BP, Stainer MC, Maynard RL. The biomechanical response of the thorax to nonpenetrating impact with particular reference to cardiac injuries. J Trauma 1982;22: 994–1008. 7143513 10.1097/00005373-198212000-00004

[32] Snow N, Richardson JD, Flint LM Jr. Myocardial contusion: implications for patients with multiple traumatic injuries. Surgery 1982;92: 744–750. 7123494

[33] Dua A, McMaster J, Desai PJ, Desai SS, Kuy S, Mata M, et al. The Association between Blunt Cardiac Injury and Isolated Sternal Fracture. Cardiol Res Pract 2014; 10.1155/2014/629687 PMC3933392 24653859

[34] Pasquale M, Fabian TC. Practice management guidelines for trauma from the Eastern Association for the Surgery of Trauma. J Trauma 1998;44: 941–956. 9637148 10.1097/00005373-199806000-00001

[35] Bergs J, Hellings J, Cleemput I, Zurel Ö, De Troyer V, Van Hiel M, et al Systematic review and meta-analysis of the effect of the World Health Organization surgical safety checklist on postoperative complications. Br J Surg 2014;101(3): 150–158 10.1002/bjs.9381 24469615

[36] Advanced Trauma Life Support for Doctors (Student Course Manual), 9th ed. Chicago: American College of Surgeons; 2012.

